# Toxicity of Engineered Nanomaterials to Microalgae: Mechanisms, Modulating Factors, Combined Effects, and Methodological Advances

**DOI:** 10.3390/molecules31122069

**Published:** 2026-06-12

**Authors:** Pengcheng Sheng, Lei Xv, Feng Lin, Yanzhou Ding, Yuchen Wang, Boyi Sun, Juyang Fu, Yunfei He, Dongren Zhou

**Affiliations:** 1 Key Laboratory of Healthy Freshwater Aquaculture, Ministry of Agriculture and Rural Affairs, Zhejiang Institute of Freshwater Fisheries (Zhejiang Freshwater Fishery Environmental Monitoring Station), Huzhou 313001, China; 2 School of Life and Environmental Sciences, Hangzhou Normal University, Hangzhou 310018, China; 3 School of Engineering, Hangzhou Normal University, Hangzhou 310018, China

**Keywords:** engineered nanomaterials, microalgae, toxicity, oxidative stress, environmental factors, combined toxicity

## Abstract

Engineered nanomaterials are widely used in environmental remediation, agriculture, and industrial applications owing to their large specific surface area, high reactivity, and tunable physicochemical properties. However, their release into aquatic environments has raised increasing concerns regarding potential risks to primary producers. Microalgae are highly sensitive to environmental stressors and play essential roles in photosynthesis, nutrient cycling, carbon fixation, and aquatic food-web stability, making them important model organisms for assessing the toxicity of engineered nanomaterials. This review summarizes the toxic effects and mechanisms of representative engineered nanomaterials, including metal and metal oxide nanoparticles, nanoplastics, and carbon-based nanomaterials, on microalgae. Major toxic pathways include nanoparticle attachment and aggregation on algal surfaces, shading effects, membrane damage, altered permeability, cellular internalization, toxic ion release, reactive oxygen species overproduction, photosynthetic inhibition, and metabolic disturbance. The review further discusses how particle size, morphology, surface coating, dissolution, aging, light, pH, and natural organic matter regulate nanomaterial bioavailability and toxicity. Combined toxicity caused by coexisting nanoparticles or emerging pollutants is also considered, with emphasis on synergistic, antagonistic, and concentration-dependent effects. Finally, recent methodological advances, such as near-native imaging, Raman-based spectroscopy, particle-specific elemental analysis, and multi-omics approaches, are highlighted. This review provides an integrated perspective for understanding nanomaterial toxicity to microalgae and supports future ecological risk assessment in aquatic environments.

## 1. Introduction

Engineered nanomaterials have been widely developed and applied in water treatment and environmental remediation because of their large specific surface area, abundant reactive sites, and excellent catalytic performance [1,2]. Representative nanomaterials, including metal and metal oxide nanoparticles, nanoplastics, and carbon-based nanomaterials, are increasingly released into aquatic environments during production, use, disposal, and aging processes [3,4,5,6,7]. Once they enter natural waters, these materials may aggregate, dissolve, sediment, age, form environmental coronas, or interact with natural organic matter and biological surfaces, thereby altering their mobility, bioavailability, and toxicity [8,9].

The ecological risks of engineered nanomaterials in aquatic systems remain difficult to predict [10]. Microalgae are primary producers in aquatic ecosystems and play essential roles in carbon fixation, oxygen production, nutrient cycling, and energy transfer [11,12]. Through photosynthesis, microalgae drive carbon and nitrogen cycling, support energy flow and material transfer, and contribute substantially to the maintenance of aquatic ecological balance [13,14]. Owing to their wide distribution, high abundance, short reproductive cycles, low nutrient requirements, and high sensitivity to environmental pollutants, microalgae are widely recognized as ideal model organisms for ecotoxicological studies and are commonly used to assess the health of aquatic ecosystems [7]. Therefore, microalgae provide a sensitive biological window for evaluating the ecological risks of engineered nanomaterials.

Nanoparticles can induce multiple toxic responses in microalgae through both extracellular interactions and intracellular disturbance [15]. On the one hand, nanoparticles may directly adhere to algal cell surfaces through electrostatic attraction, surface charge interactions, or heterogeneous aggregation, resulting in membrane damage, altered membrane permeability, and impaired nutrient exchange [4,16]. On the other hand, some nanoparticles can cross the algal cell barrier and enter cells, where they disrupt the structural integrity of organelles such as chloroplasts and mitochondria and may further damage DNA [17]. In addition, many nanoparticles possess membrane-penetrating ability and photoactivity, which can aggravate their adverse effects on microalgal cells under certain environmental conditions [18]. Once internalized or closely attached to algal cells, nanoparticles can promote the excessive generation of reactive oxygen species (ROS), leading to lipid peroxidation, oxidative stress, inhibition of photosynthesis, and disruption of membrane transport functions [19]. Under severe nanoparticle stress, algal cells may also release intracellular organic matter and toxic metabolites, thereby posing secondary risks to aquatic organisms and water quality [5,20]. For example, Tang et al. reported that exposure to zinc oxide nanoparticles (ZnO NPs) above a certain concentration could damage algal cells and promote the release of cellular organic matter, which may further influence microcystin production and transformation [21]. Moreover, nanoparticles can accumulate in algal cells, making microalgae an important entry point for nanoparticle transfer in aquatic food webs. Previous studies have shown that algal cells can accumulate cadmium-containing nanoparticles [22], and that Ag NP aggregates associated with algal cells increase with exposure time, leading to enhanced cellular accumulation [23]. Once incorporated into algal biomass, these nanoparticles may be transferred to higher trophic levels through grazing, potentially affecting trophic interactions and increasing long-term ecological risks.

To improve the transparency and reproducibility of this review, a literature search was conducted using Web of Science and Google Scholar. The search terms included combinations of “engineered nanomaterials”, “nanoparticles”, “nanoplastics”, “carbon-based nanomaterials”, “microalgae”, “algal toxicity”. Peer-reviewed articles were preferentially selected when they reported nanomaterial properties, algal species, exposure conditions, toxic endpoints, or mechanistic information. Studies lacking sufficient information on exposure conditions, material characterization, or biological endpoints were not used as major evidence. Additional relevant references were identified from the reference lists of selected articles.

This review summarizes the toxic effects and mechanisms of representative engineered nanomaterials on microalgae, with emphasis on metal and metal oxide nanoparticles, nanoplastics, and carbon-based nanomaterials. It further discusses how physicochemical properties and environmental factors regulate toxicity, evaluates combined toxicity under realistic exposure scenarios, and highlights emerging methods for cross-scale characterization of nanomaterial-microalgae interactions. By integrating material behavior, biological responses, and environmental modulation, this review aims to provide a clearer framework for understanding nanomaterial-induced toxicity and improving ecological risk assessment in aquatic systems.

## 2. Toxic Effects and Mechanisms of Engineered Nanomaterials in Microalgae

Engineered nanoparticles exhibit diverse toxic effects on microalgae depending on their composition, size, morphology, surface properties, and environmental behavior. Although different nanomaterials have distinct toxic characteristics, several common responses can be identified, including growth inhibition, chlorophyll reduction, photosynthetic impairment, oxidative stress, membrane damage, cellular internalization, and metabolic disturbance. Based on material composition and structural characteristics, this section discusses three major classes of engineered nanomaterials: metal and metal oxide nanoparticles, nanoplastics, and carbon-based nanomaterials.

### 2.1. Metal and Metal Oxide Nanoparticles

Metal and metal oxide nanoparticles are among the most extensively investigated engineered nanomaterials, owing to their broad application potential in environmental engineering [24]. As summarized in Table 1, their toxic effects on microalgae are mainly reflected in growth inhibition, reduced chlorophyll synthesis, impaired photosynthetic performance, and increased cellular damage. The severity of these effects is strongly dependent on algal species, nanoparticle type, exposure dose, and exposure duration.

Among these materials, silver nanoparticles (Ag NPs) have attracted considerable attention because of their strong antibacterial activity and catalytic properties [25,26]. However, Ag NPs can exert pronounced toxic effects on microalgae, including changes in cell membrane fluidity, disruption of cellular structure, oxidative stress, and photosynthetic inhibition [27,28,29,30]. For example, exposure of *Chlorella pyrenoidosa* to 10 mg/L Ag NPs for 96 h caused approximately 50% growth inhibition and a 75% reduction in chlorophyll yield, indicating severe impairment of both cell proliferation and photosynthetic pigment synthesis [31]. In *Chlamydomonas reinhardtii*, Ag NPs increased membrane permeability and promoted nanoparticle internalization, ultimately leading to chloroplast damage and irreversible cellular injury associated with excessive ROS production [30].

Recent transcriptomic and metabolomic studies have further revealed molecular-level responses of microalgae to Ag NPs exposure. Ag NPs reduced algal cell size and granularity, activated the ascorbate-glutathione (AsA-GSH) antioxidant pathway to alleviate ROS stress, and downregulated most genes involved in photosynthetic systems, suggesting impaired photosynthetic activity at the transcriptional level [32]. In addition, metabolic pathways related to starch and sucrose metabolism and amino acid metabolism were inhibited, resulting in reduced physiological activity of algal cells [3,33]. The toxicity of Ag NPs is also morphology-dependent. Silver nanospheres (AgNSs), nanocubes (AgNCs), and nanoplates (AgPLs) caused different degrees of subcellular damage in *Chlorella vulgaris* and *Scenedesmus obliquus*, with AgPLs showing the strongest growth inhibition. This stronger toxicity may be associated with their higher colloidal stability and morphology-dependent Ag accumulation, while *Scenedesmus obliquus* was more sensitive than *Chlorella vulgaris* [34].

Titanium dioxide nanoparticles (TiO_2_ NPs) are another representative class of metal oxide nanoparticles with broad use in photocatalysis, coatings, and water treatment. Their toxicity to microalgae is mainly associated with cellular uptake, interfacial interactions, oxidative stress, and photosynthetic disturbance. TiO_2_ NPs can enter algal cells through energy-dependent endocytosis or other uptake pathways, thereby disturbing intracellular homeostasis and inhibiting algal growth. In *Chlorella* sp., anatase TiO_2_ NPs at 0.5 mg/L caused approximately 27.2% growth inhibition and a 17.2% reduction in chlorophyll yield after 72 h [35]. In *Microcystis aeruginosa*, exposure to 100 mg/L TiO_2_ NPs for 48 h resulted in more than 54% growth inhibition [36], while prolonged exposure of *Chlorella minutissima* to 50 mg/L TiO_2_ NPs led to complete growth inhibition and a 50% decrease in chlorophyll yield [37]. These results indicate that TiO_2_ NPs toxicity is strongly dependent on exposure level and duration. Moreover, extracellular polymeric substances (EPS) can promote heteroaggregation between TiO_2_ NPs and algal cells, leading to nanoparticle accumulation on the cell surface and altered bioavailability [38].

Zinc oxide nanoparticles (ZnO NPs) produced greater toxicity to *Coelastrella terrestris* than bulk ZnO, mainly due to their higher bioavailability, surface adsorption, internalization, and ion release [39]. Exposure to ZnO NPs can induce oxidative stress, morphological shrinkage, pigment loss, and reduced cell viability. For example, 10 mg/L ZnO NPs caused up to 78% growth inhibition in *Skeletonema costatum* after 96 h [40], while 200 mg/L ZnO NPs reduced chlorophyll yield by 92.5% and inhibited the growth of *S. platensis* by 87.3% after 96 h [41]. These findings indicate that ZnO NP toxicity involves both particle-specific effects and dissolved Zn-mediated toxicity. At relatively high concentrations, ZnO NPs may also damage cell integrity and promote the release of intracellular organic matter into the surrounding medium [42,43].

Cu-based nanoparticles, including Cu and CuO NPs, have been widely used in catalysis, sensors, and other engineering applications [44,45,46]. Due to their smaller particle size and high reactivity, Cu-based NPs can adhere to algal cell surfaces, damage membrane structures, enter cells, and trigger oxidative stress and lipid peroxidation [47,48]. In *Microcystis aeruginosa*, CuO NPs inhibited growth by inducing ROS accumulation, altering mitochondrial potential, and causing severe mitochondrial damage [49]. More recent evidence showed that copper hydroxide-based nanopesticides at 10 mg/L reduced *M. aeruginosa* proliferation by 88.3% after 7 days, accompanied by a 94.1% reduction in chlorophyll yield, membrane disruption, and suppression of ATP production [7]. In addition, Cu NP exposure can alter secondary metabolites, including increased phenolic compounds and decreased carotenoid levels [50].

Iron oxide nanoparticles, especially Fe_3_O_4_ and γ-Fe_2_O_3_, are important metal oxide nanomaterials widely used in adsorption, water treatment, environmental remediation, and magnetic separation [51,52,53]. Their interactions with microalgae involve surface attachment, aggregation with algal cells or extracellular polymeric substances. Compared with Ag or ZnO NPs, iron oxide nanoparticles generally show weaker ion-release toxicity, but their aggregation and surface transformation can still affect light capture, membrane integrity, and photosynthetic activity [54]. Because of their magnetic properties, Fe_3_O_4_ and γ-Fe_2_O_3_ are also used for algae harvesting, pollutant removal, and magnetic recovery [55]; however, these applications may increase direct contact between particles and algal cells. Therefore, both their environmental application value and potential ecological risks should be considered.

**Figure 1:**
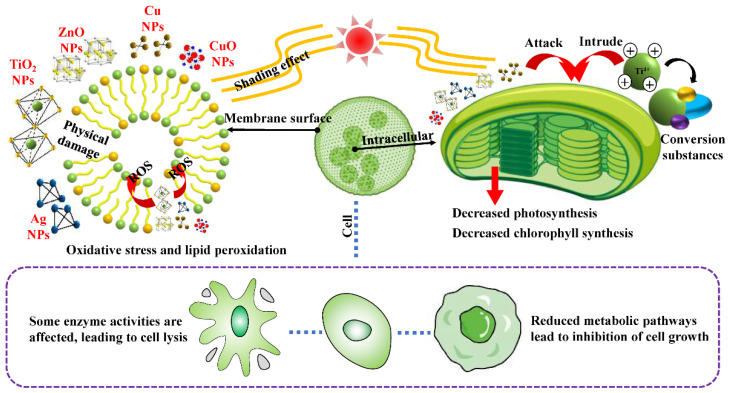
Mechanisms of toxicity of some metal and metal oxide NPs to algae.

As presented in Figure 1, metal and metal oxide nanoparticles, including Ag, TiO_2_, ZnO, Cu/CuO, and iron oxide nanoparticles, can impair microalgae through multiple interconnected pathways. Some nanoparticles attach to the cell surface and reduce light capture through shading effects, thereby weakening photosynthesis. Others damage membrane structures, increase permeability, and enter algal cells, leading to oxidative stress and intracellular injury. Released toxic ions may further disrupt photosystem structures and inhibit chlorophyll biosynthesis. Consequently, algal cells may undergo deformation, shrinkage, structural degradation, and eventual loss of viability.

Overall, metal and metal oxide nanoparticles do not follow a single toxicity pattern. Ag and ZnO NPs often show strong toxicity through both particle-specific effects and dissolved metal ions, whereas TiO_2_ toxicity is more dependent on light, aggregation, and surface attachment. Therefore, toxicity should not be compared only by nominal concentration; algal species, exposure duration, particle transformation, dissolved ions, and endpoints should also be considered.

**Table 1:** Effects of selected nanometals or metal oxides on growth inhibition aspects of specified algae.

NPs	Algae	Exposure Time	Dose (mg/L)	EC50 (mg/L)	Reduction in Chlorophyll Yield (%)	Growth Inhibition (%)	Ref.
Ag NPs	*Chlorella pyrenoidosa*	96 h	10	15.99	75	50	[31]
Ag NPs	*Chlorella vulgaris*	96 h	0.09	0.11		22	[56]
Ag NPs	*Skeletonema costatum*	24 h	5	25.77	2.7	9.5	[26]
Ag NPs	*Chlorella vulgaris*	96 h	100	-	92	91.1	[57]
Ag NPs	*Chlorella vulgaris*	168 h	0.5	-	-	58	[34]
	*Scenedesmus obliquus*	168 h	5	-	-	76	
TiO_2_ NPs (Anatase)	*Chlorella* sp.	72 h	0.5	3.4	17.2	27.2	[35]
TiO_2_ NPs	*Microcystis aeruginosa*	48 h	100	-	-	54.1	[36]
TiO_2_ NPs	*Chlorella minutissima*	720 h	50	-	50	100	[37]
ZnO NPs	*Skeletonema costatum*	96 h	10	3.6	-	<78	[40]
ZnO NPs	*Tetraselmis suecica*	72 h	1	3.91	-	-	[58]
ZnO NPs	*Spirulina platensis*	96 h	200	31.56	92.5	87.3	[41]
CuNPs	*Microcystis aeruginosa*	168 h	10	-	94.1	88.3	[7]
iron oxide nanoparticles	*Chlorella* sp.	168 h	20	-	25.80	37.02	[54]
iron oxide nanoparticles	*Coelastrella terrestris*	600 h	50	-	-	33.33	[59]

### 2.2. Nanoplastics

Nanoplastics are characterized by high stability, slow degradation, small particle size, and strong permeability, which enable them to cross biological barriers and be taken up by microalgal cells [60]. Compared with microplastics, nanoplastics generally exhibit higher bioavailability and toxicity, and are therefore regarded as one of the most concerning forms of marine litter [61]. As summarized in Table 2, the toxic effects of engineering-related nanoplastics on microalgae are typically dose- and time-dependent, mainly including growth inhibition, reduced chlorophyll synthesis, oxidative stress, and cellular damage.

Polystyrene nanoplastics are the most frequently investigated nanoplastic type in microalgal toxicity studies. For example, exposure of *Chlamydomonas reinhardtii* to 500 mg/L polystyrene microbeads for 96 h resulted in 61.7% growth inhibition, a 72.8% reduction in chlorophyll yield, and a 98.7% increase in ROS production [20]. Similarly, 500 mg/L polystyrene nanoplastics inhibited the growth of *Chlorella pyrenoidosa* by 72.8% and reduced chlorophyll yield by 37.6% after 96 h [62]. Even at lower concentrations, nanoplastics can exert marked effects; for instance, fluorescent nanoplastics at 1 mg/L caused approximately 50% growth inhibition in *Scenedesmus obliquus* after 72 h [63]. In *Microcystis aeruginosa*, 5 mg/L polystyrene exposure caused less than 25% growth inhibition but reduced chlorophyll yield by approximately 27.2%, suggesting that photosynthetic pigments may be more sensitive than growth endpoints under certain exposure conditions [64].

Microscopic observations provide direct evidence for nanoplastic-induced structural damage. Nigam et al. observed slight deformation of the cell wall of *C. pyrenoidosa* under high-concentration nanoplastic exposure, which was associated with nanoplastic internalization [62]. Yan et al. further reported that polystyrene nanoplastics increased membrane permeability in *C. reinhardtii*, thereby accelerating nanoplastic internalization. The internalized particles were observed to be encapsulated in intracellular vesicles [20]. These results suggest that nanoplastics can impair membrane integrity and promote cellular uptake. Increased ROS and MDA levels further indicate that oxidative stress and lipid peroxidation contribute to membrane damage and enhanced internalization [65].

The toxicity of nanoplastics to algae is also attributed to ROS overproduction, membrane disruption, mitochondrial dysfunction, and interference with the cell division cycle. Oxidative stress may also stimulate the secretion of extracellular polymeric substances and enhance antioxidant enzyme activities, which can serve as defense responses against nanoplastic-induced stress. In addition, electrostatic attraction between positively charged nanoplastics and negatively charged algal cell membranes can promote the accumulation of nanoplastics on the algal cell surface [66]. Overall, nanoplastic exposure disrupts normal physiological and biochemical processes in microalgae by reducing nutrient uptake, inhibiting photosynthesis, inducing oxidative stress, causing physical damage, and promoting homo- or hetero-aggregation between nanoplastics and algal cells [67].

These studies indicate that nanoplastic toxicity is strongly affected by particle size, surface charge, concentration, and exposure time. High-dose studies help identify mechanisms such as ROS generation, membrane damage, and internalization, but they may overestimate ecological risk. Low-dose studies are more environmentally relevant but require longer exposure and more sensitive endpoints.

**Table 2:** Effects of some nanoplastics on algae indicators.

Type	Algae	Exposure Time (h)	Dose (mg/L)	Growth Inhibition (%)	Reduction in Chlorophyll Yield (%)	ROS Yield (%)	Ref.
polystyrene nanoplastics (+IBU)	*Chlorella pyrenoidosa*	96	1	63.9	-	-	[63]
polystyrene microbeads	*Chlamydomonas reinhardtii*	96	500	61.7	72.8	98.7	[20]
polystyrene	*Gymnodinium aeruginosum*	96	75	63.7	-	700	[68]
polymethyl methacrylate		72		21.4		200	
polystyrene	*C.pyrenoidosa*	96	50	10.53	29.64	<254.4	[60]
polystyrene	*Chlorella pyrenoidosa*	96	500	72.8	37.6	-	[62]
fluorescent nanoplastics	*Scenedesmus obliquus*	72	1	50	-	<170	[69]
polystyrene	*Microcystis aeruginosa*	96	5	<25	≈27.2	-	[64]
polystyrene	*Algal cell membrane*	3	100		15	<25	[70]
palladium-doped polystyrene	*Anabaena*	72	200	151.3	-	300	[71]
	*Chlamydomonas reinhardtii*			247.8		53	

### 2.3. Carbon-Based Nanomaterials

Carbon-based nanomaterials have attracted broad attention due to their high stability, mechanical flexibility and diverse applications in antimicrobial, environmental, and engineering fields. The major carbon-based nanomaterials include graphene-family materials (GFMs) [72,73,74,75], carbon nanotubes (CNTs) [76,77], and carbon nanofibers (CNFs) [78]. As shown in Table 3, these materials can inhibit microalgal growth and photosynthetic activity through direct physical damage, oxidative stress, shading effects, nutrient depletion, and aggregation with algal cells.

Graphene-based nanomaterials, especially graphene oxide (GO) and reduced graphene oxide (rGO), can interact strongly with algal cell surfaces. Owing to their sharp edges and sheet-like structures, rGO and related materials may damage the cell wall and membrane, facilitate cellular penetration, and induce intracellular oxidative stress [79,80]. Under oxidative and osmotic stress, membrane integrity can be further disrupted, resulting in leakage of intracellular components, including DNA fragments and K^+^ ions involved in osmotic regulation. In *Chlorella pyrenoidosa*, exposure to 50 mg/L GO for 96 h caused 83% growth inhibition, while rGO and multilayer graphene caused even higher or comparable inhibition, reaching 92% and 77%, respectively [75]. These results indicate that structural differences among graphene derivatives can lead to distinct toxic responses. GO may also exert a shading effect, reducing light availability and further inhibiting algal growth and biochemical activity. In addition, graphene-family materials can adsorb nutrients such as N, P, Mg, and Ca from the culture medium, resulting in nutrient depletion and indirect inhibition of algal physiological processes [75]. Xin et al. also reported that GO promoted the release of dissolved organic matter and algal toxins from algal cells in a concentration-dependent manner [81].

CNTs can readily bind to algal cells or algal-derived metabolites, promoting aggregate formation and adhesion to the algal surface [82]. Their toxicity is also influenced by the presence of residual or doped heavy metals [83]. Exposure of *Heterosigma akashiwo* to CNTs at high concentrations (>10 mg/L) significantly reduced algal cell abundance and disrupted cellular integrity [84]. This effect may be associated with enhanced aggregation at higher CNT concentrations, which increases physical contact and toxic stress. In *Chlorella vulgaris*, CNT exposure at 50 mg/L for 96 h caused 61% growth inhibition [85], while another study reported that CNT exposure reduced growth and chlorophyll yield by 61.7% and 67.3%, respectively [86]. Wu et al. further reported that high concentrations of single-walled carbon nanotubes (SWCNTs; >5.00 mg/L) inhibited the growth of *Microcystis aeruginosa* in a dose-dependent manner, with inhibition exceeding 90% at 100 mg/L [87]. These inhibitory effects were mainly associated with reduced antioxidant enzyme activity, enhanced lipid peroxidation, disruption of cellular metabolism and photosynthetic systems, and decreased contents of photosynthetic pigments, soluble sugars, and proteins.

CNFs mainly affect algal cells through direct mechanical damage. CNF exposure can disrupt cellular integrity and induce irregular morphological changes, which are often more pronounced than those caused by CNTs or graphene-based materials. In *Klebsormidium flaccidum*, exposure to 0.1 mg/L CNFs for 96 h caused 64% growth inhibition [78], indicating that fibrous carbon nanomaterials can exert strong toxicity even at relatively low concentrations. Metal impurities in CNFs may further aggravate algal deformation and cellular injury [84]. Munk et al. suggested that CNF-induced growth inhibition and morphological changes are mainly driven by direct mechanical damage and oxidative stress [78]. In addition to CNTs, CNFs, and graphene-based materials, functionalized carbon nanomaterials, such as carboxyl-modified graphene and amine-modified graphene, can also inhibit microalgal growth and photosynthetic activity [73].

Carbon-based nanomaterials should not be treated as a homogeneous group. Graphene-family materials mainly act through sheet-edge damage, shading, nutrient adsorption, and oxidative stress, whereas CNTs and CNFs may cause stronger mechanical disturbance due to their fibrous morphology. Their toxicity should therefore be interpreted according to dimensionality, surface chemistry, impurities, aggregation, and contact with algal cells.

**Table 3:** Effects of some carbon-based nanomaterials on algae.

Type	*Algae*	Exposure Time (h)	Dose (mg/L)	Growth Inhibition (%)	Reduction in Chlorophyll Yield (%)	Ref.
CNTs	*Chlorella vulgaris*	96	50	61 ± 7	-	[85]
CNTs	*Chlorella vulgaris*	72	0.05	61.7	67.3 ± 3	[86]
CNFs				48.83	52.2 ± 1	
CNFs	*Klebsormidium flaccidum*	96	0.1	64	-	[78]
CNTs	*Pseudokirchneriella subcapitata*	72	6.1	20~30	33	[88]
GO	*Chlorella pyrenoidosa*	96	50	83	-	[75]
rGO				92		
MG				77		
CNTs	*Chlamydomonas reinhardtii*	7 × 24	5	24.3	54.45	[77]
CNTs	*Skeletonema costatum*	96	1	58	98	[89]
G	*Scenedesmus obliquus*	72	80	99.9	97	[73]
GO				31.8	96	
G-COOH				23.7	86	
G-NH_2_				16.4	41	

Note: MG, G, GO, G-COOH, and G-NH_2_ denote multilayer graphene, graphene, graphene oxide, carboxyl-modified graphene, and amine-modified graphene, respectively.

## 3. Modulating Effects of Physicochemical Properties and Environmental Conditions

**Figure 2:**
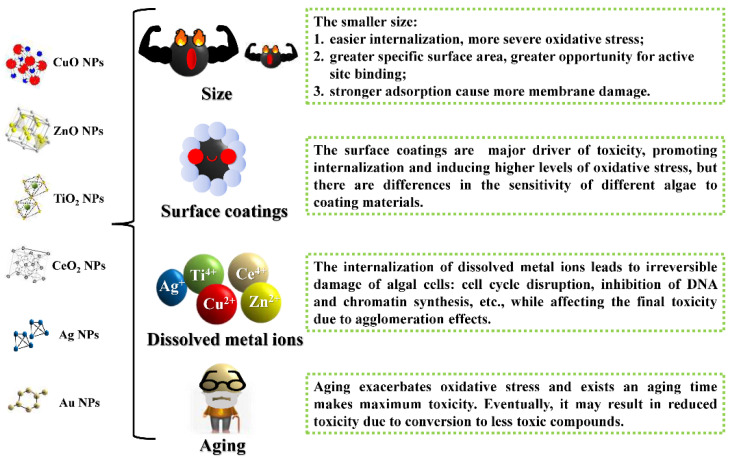
Effects of physicochemical characteristics of NPs on the toxic effects of algae.

The toxicity of engineered nanomaterials to microalgae is not determined only by their chemical composition, but also by their physicochemical properties and environmental transformation. As illustrated in Figure 2, intrinsic properties such as particle size, morphology, surface coating, dissolution behavior, and aging can regulate aggregation, surface attachment, cellular uptake, ion release, and bioavailability. Meanwhile, environmental factors, including light, pH, and natural organic matter, further modify nano-bio interfacial interactions and ultimately determine whether toxicity is enhanced or mitigated.

### 3.1. Physicochemical Characteristics of Nanoparticles

#### 3.1.1. Size and Morphology

Particle size is one of the most important factors regulating nanomaterial toxicity to microalgae. Smaller nanoparticles generally have larger specific surface areas, higher reactivity, and stronger potential for cellular attachment or internalization. As a result, they are more likely to induce oxidative stress, lipid peroxidation, photosynthetic inhibition, and growth suppression. For example, smaller polystyrene particles caused stronger toxic effects than larger particles, including increased ROS production and reduced chlorophyll a content, photosynthetic yield, and enzymatic activities [70]. Similarly, when *Pseudokirchneriella subcapitata* was exposed to TiO_2_ NPs, primary particles with smaller sizes induced stronger growth inhibition and lipid peroxidation than larger secondary aggregates [90].

However, size-dependent toxicity is often coupled with particle aggregation and morphology. In aqueous media, nanoparticles rarely remain as isolated primary particles; instead, they form aggregates with different hydrodynamic diameters, surface charges, and settling behaviors. Therefore, the apparent particle size in exposure systems may differ substantially from the primary size reported by manufacturers. Morphology can further modulate these effects. Spherical ZnO NPs caused stronger damage than rod-like ZnO NPs, while the toxicity of rod-like ZnO NPs decreased with increasing size [91]. For Au engineered nanoparticles, only 10 nm spherical particles and 10 × 45 nm rod-shaped particles caused obvious membrane damage, suggesting that both size and shape regulate particle attachment and physical stress on algal cells [92]. Overall, particle size and morphology jointly influence nanomaterial toxicity by regulating surface reactivity, aggregation behavior, contact probability with algal cells, and internalization potential [93].

#### 3.1.2. Surface Coating

Surface coating strongly influences nanoparticle stability, surface charge, dissolution, aggregation, and biological interactions. Coatings may either enhance or reduce toxicity depending on their chemical properties and their effects on particle-cell interactions. For example, Ag NPs with different coatings may show similar toxic effects when the released Ag^+^ concentration is comparable, suggesting that both coating properties and ion release should be considered when interpreting toxicity [85]. Chen et al. also showed that coating type, such as carboxymethyl cellulose and chitosan, was a key factor controlling the toxicity of selenium nanoparticles [94].

In many cases, surface coatings modify toxicity by altering nanoparticle internalization and ROS generation. Polyvinylpyrrolidone-coated CeO_2_ NPs promoted internalization in *Chlamydomonas reinhardtii* and increased ROS formation, whereas uncoated CeO_2_ NPs mainly caused membrane damage through direct contact with cells [95]. Surfactant coatings such as sodium dodecyl benzene sulfonate and octylphenoxyethanol also promoted the internalization of multi-walled carbon nanotubes in *Chlorella* cells and enhanced oxidative stress. In contrast, humic acid coatings can reduce toxicity by changing membrane permeability, limiting particle internalization, and suppressing ROS production [96]. These findings indicate that surface coatings should not be regarded simply as stabilizers; rather, they can reshape nano-bio interactions by modifying particle dispersion, surface charge, corona formation, and cellular uptake. In addition, algal species differ in their sensitivity to the same coated nanoparticles, reflecting species-specific differences in cell wall composition, EPS secretion, and surface binding capacity [97].

#### 3.1.3. Dissolution and Metal Ion Release

For metal and metal oxide nanoparticles, dissolution and toxic ion release are major determinants of algal toxicity. Released ions can cross damaged or permeable membranes, disturb intracellular metal homeostasis, inhibit photosynthesis, and promote oxidative stress. The toxicity of Ag NPs, for example, is closely related to Ag^+^ release, although particulate effects such as surface attachment and internalization may also contribute. Xu et al. reported time- and dose-dependent effects of Ag NPs on *Chlamydomonas reinhardtii*, including decreased growth rate and photosynthetic metabolites, increased MDA content, and disruption of antioxidant defense [28,29,91,98]. Low concentrations of Ag NPs or Ag^+^ may perturb antioxidant defense and nitrogen metabolism, whereas obvious growth inhibition generally occurs at higher exposure levels [4].

Ion-mediated toxicity is also influenced by particle size, aggregation, and algal density. Smaller Ag NPs often show stronger toxic effects because their larger surface area favors dissolution and biological interaction [99]. However, aggregation may reduce the concentration of bioavailable dissolved ions and limit direct contact with cells. In dense algal cultures, extracellular polysaccharides and dissolved organic carbon can bind Ag NPs or Ag^+^, thereby reducing particle availability and cellular internalization [23].

#### 3.1.4. Time-Dependent Transformation and Degradation of Nanomaterials

Nanoparticles inevitably undergo aging and transformation in aquatic environments, including oxidation, sulfidation, phototransformation, dissolution, aggregation, and adsorption of natural organic matter. These processes can change particle size distribution, surface chemistry, dissolution behavior, and bioavailability, thereby altering toxicity. For Ag NPs, aged particles may induce stronger lipid peroxidation than fresh particles, partly because aging can modify ROS generation, coating stability, and ion release [100]. The stability of aged Ag NPs also depends on coating materials. For example, curcumin-coated aged Ag NPs were more stable than those coated with tyrosine or epigallocatechin gallate, and less stable coatings promoted aggregation and reduced surface reactivity [101].

However, aging does not always lead to stronger toxicity. Its effects are often non-linear and material-specific. In some cases, short-term aging may increase toxicity by enhancing dissolution or surface reactivity, whereas longer aging may reduce toxicity because particles aggregate, settle, or transform into less bioavailable species. For example, *P. subcapitata* exposed to aged CeO_2_ NPs showed the strongest toxicity after 3 days of aging rather than after longer aging periods [102]. In other cases, ZnO NPs aged for 30 days caused stronger effects on *Chlorella vulgaris* [40]. These results suggest that there may be a critical aging window during which nanoparticle toxicity reaches a maximum. Therefore, aging should be treated as a dynamic transformation process rather than a unidirectional increase or decrease in toxicity.

### 3.2. Environmental Factors

#### 3.2.1. Light

Light is a key environmental factor regulating the toxicity of photoactive nanoparticles. Many metal oxide nanoparticles, such as TiO_2_ and ZnO NPs, can generate ROS under UV or visible light, thereby enhancing oxidative stress and membrane damage in microalgae. ZnO NPs showed stronger toxicity under UV-C irradiation, accompanied by changes in nanoparticle accumulation, increased antioxidant enzyme activities, enhanced lipid peroxidation, and reduced algal activity [103]. Under UV-A irradiation, photoactive nanoparticles may undergo phototransformation and changes in hydrodynamic size, which can further influence their internalization and toxicity [104].

Light can also affect algal defense responses. Increased radiation may reduce EPS secretion by *Chlorella*, weakening the protective barrier around algal cells and increasing nanoparticle internalization [105]. For TiO_2_ NPs, photocatalytic reactions can promote oxidation of functional groups on algal cell surfaces, including C-N, C=O, C-O-C, and P=O groups, leading to cell wall and membrane disruption [106]. The sensitivity of algae to light-modulated nanoparticle toxicity varies among species and environments. Marine microalgae such as *Phaeodactylum tricornutum* may show stronger responses under UV-A exposure than freshwater microalgae such as *Chlamydomonas reinhardtii*, including more severe membrane damage and reduced EPS production [5]. Thus, light does not simply enhance toxicity; it regulates toxicity through combined effects on nanoparticle photochemistry, algal physiology, EPS secretion, and ROS balance.

#### 3.2.2. pH Value

pH affects nanoparticle toxicity by altering surface charge, aggregation behavior, dissolution rate, metal ion speciation, and algal cell surface properties. The pH effect is often concentration-dependent. For alumina-coated SiO_2_ NPs, low-dose exposure did not show clear pH-dependent toxicity, whereas high-dose exposure produced stronger toxicity at pH 6.0–6.8 [107]. This effect may be related to changes in surface charge and aggregation, which influence the contact between nanoparticles and algal cells.

pH can also regulate the toxicity of Ag NPs. In *Microcystis aeruginosa*, Ag NPs caused stronger growth inhibition under low-pH conditions, likely because pH affected particle size, Ag^+^ activity, and nanoparticle stability [26]. In addition, different nanoparticles show different pH-dependent heteroaggregation behaviors with algal cells. Rutile TiO_2_ and *β*-Al_2_O_3_ showed weak heteroaggregation and were relatively insensitive to pH changes, whereas microporous SiO_2_ preferentially heteroaggregated under acidic conditions, and rutile TiO_2_ showed more obvious heteroaggregation under neutral conditions [108].

#### 3.2.3. Natural Organic Matter

Natural organic matter (NOM), including humic substances, fulvic acids, proteins, polysaccharides, and algal-derived extracellular polymers, plays a dual role in nanoparticle toxicity. On the one hand, NOM can increase nanoparticle dispersion, alter surface charge, promote cellular internalization, or facilitate transformation into more toxic species. For example, dissolved organic matter enhanced the toxicity of Al_2_O_3_ NPs to *Scenedesmus obliquus*, as reflected by increased ROS levels, elevated SOD activity, mitochondrial membrane potential loss, and increased membrane permeability [109]. Suwannee River fulvic acid also increased the cellular accumulation of CuO NPs in *Microcystis aeruginosa* and promoted transformation into more toxic Cu_2_O, thereby aggravating growth inhibition [110].

In addition, the relative growth rate of algal cells was reduced and reactive oxygen species were increased in a dose-dependent manner through the activation of related enzymes and peptides [111]. When the level of oxidative stress reaches a certain threshold, it triggers the algal cell’s defense mechanism to secrete a certain amount of EPS, but EPS is potentially toxic and reduces the bioavailability of ENPs, which further enhances the toxic effects [112].

On the other hand, NOM can reduce nanoparticle toxicity by forming surface coronas, increasing electrostatic repulsion, blocking active sites, and limiting direct particle-cell contact. Humic acid (HA) can weaken the adhesion of nanoparticles to algal cells and reduce intracellular ROS production, thereby mitigating toxicity [74]. It can also regulate algal metabolic responses, such as glutathione metabolism and alanine biosynthesis, to alleviate oxidative stress-related cell wall damage. For graphene-family materials, humic acid reduced toxicity by decreasing contact with algal cells, limiting particle deposition on cell surfaces, and scavenging ROS [113]. Therefore, the role of NOM is context-dependent: it may either enhance or mitigate toxicity depending on NOM type, concentration, molecular composition, nanoparticle properties, and algal species.

## 4. Combined Toxicity Under Environmentally Realistic Exposure Scenarios

In natural aquatic environments, engineered nanomaterials rarely occur as isolated stressors. Instead, they often coexist with other nanoparticles, natural colloids, disinfectants, pharmaceuticals, antibiotics, pesticides, and organic pollutants. These coexisting substances can modify nanoparticle aggregation, dissolution, surface adsorption, cellular uptake, EPS secretion, and ROS generation, thereby leading to synergistic, antagonistic, additive, or concentration-dependent toxic effects. Therefore, combined toxicity should be considered as a dynamic outcome of interactions among nanomaterials, co-contaminants, environmental conditions, and algal physiological responses.

### 4.1. Joint Action of Multiple Nanoparticles

The co-occurrence of different nanoparticles may enhance microalgal toxicity by increasing oxidative stress, membrane damage, or intracellular metabolic disturbance. For example, exposure of *Chlorella pyrenoidosa* to a mixed system of hematite nanoparticles and Ag NPs caused more severe membrane damage, ROS overproduction, and inhibition of starch and sucrose metabolism, amino acid metabolism, and biosynthetic enzyme-related pathways [33]. This enhanced toxicity was attributed to the formation of Ag NP/hematite NP composites, which improved charge separation and promoted the generation of highly reactive free radicals.

However, combined exposure to multiple nanoparticles does not always result in stronger toxicity. In some cases, nanoparticle aggregation or deposition can reduce bioavailability and cellular internalization, thereby weakening toxic effects. For instance, with increasing exposure time, aggregation among nanoparticles may reduce the concentration of bioavailable particles or dissolved ions, and the formation of surface coatings on aggregates may further limit direct contact with algal cells [114]. Similarly, when *dinoflagellate Gymnodinium* was exposed to ZnO NPs and graphene quantum dots, the two nanoparticles adsorbed onto algal surfaces and promoted aggregation and deposition, which reduced the availability of active sites and ultimately weakened the combined toxic response [115].

The contribution of each nanoparticle to combined toxicity can also differ substantially. When *Scenedesmus obliquus* was co-exposed to ZnO NPs and CuO NPs, CuO NPs contributed more strongly to the overall toxic effect than ZnO NPs, and the combined toxicity showed dose-dependent characteristics [116]. Such differences may arise from variations in chemical composition, dissolution behavior, surface reactivity, and binding affinity with algal cell wall components. Therefore, joint nanoparticle toxicity should be interpreted not only by total exposure concentration, but also by the relative contribution, transformation, and bioavailability of each nanoparticle component.

### 4.2. Combined Toxicity of Nanoparticles and Emerging Pollutants

Nanoparticles also coexist with emerging pollutants, such as pharmaceuticals, antibiotics, pesticides, disinfectants, and persistent organic pollutants. These pollutants may alter nanoparticle behavior and algal responses by changing particle adsorption, dissolution, cellular uptake, and oxidative stress. For example, ibuprofen co-exposure with nanoplastics reduced ROS and MDA levels, alleviated growth inhibition, and decreased the bioaccumulation of both ibuprofen and nanoplastics in *Chlorella pyrenoidosa*, possibly by promoting ibuprofen biodegradation and reducing bioavailability [117]. This case indicates that nanoplastics do not necessarily enhance the toxicity of coexisting organic pollutants; instead, their effects depend on pollutant transformation and algal metabolic responses.

Photocatalytically active nanoparticles, such as TiO_2_ NPs, may interact strongly with antibiotics and other organic pollutants under light exposure. Under co-exposure conditions, algal membrane damage and nanoparticle internalization can be intensified, and the degree of growth inhibition may vary with radiation pretreatment and exposure concentration [118]. In addition, TiO_2_ NPs can show different combined effects with different organochlorine pollutants. Co-exposure with atrazine enhanced algal damage, whereas co-exposure with 3,3,4,4-tetrachlorobiphenyl reduced the co-toxicity [119]. These contrasting responses may be related to differences in pollutant adsorption onto TiO_2_ NPs, which influence nanoparticle attachment to algal surfaces and subsequent internalization.

Disinfectants and chlorinated compounds may further modify nanoparticle toxicity by promoting chemical transformation. For example, co-exposure of Ag NPs and NaOCl increased Ag NP dissolution and Ag^+^ release over time, which enhanced damage to *Microcystis aeruginosa* cell membranes and promoted toxin release [120]. This suggests that nanoparticle toxicity in treated or disinfected waters may differ substantially from that observed in single-particle laboratory exposure systems.

### 4.3. Key Determinants of Synergistic and Antagonistic Effects

Overall, combined toxicity depends on whether coexisting stressors increase or decrease nanoparticle bioavailability and cellular stress. Synergistic effects are more likely to occur when co-exposure enhances nanoparticle dissolution, ROS generation, membrane permeability, or cellular uptake. In contrast, antagonistic effects may arise when aggregation, surface coating, pollutant adsorption, or EPS-mediated binding reduces direct contact between nanoparticles and algal cells. The same combination may even shift from antagonistic to synergistic under different exposure concentrations, ratios, light conditions, pH values, or algal growth stages. Therefore, future studies should move beyond single-pollutant toxicity assays and develop environmentally realistic co-exposure models that integrate particle transformation, pollutant interactions, algal defense responses, and time-dependent toxicity.

### 4.4. Ecological Relevance of Exposure Concentrations

A major limitation of current microalgal toxicity studies is the gap between laboratory exposure concentrations and environmentally relevant levels. Many studies use mg/L-level concentrations to obtain clear toxic responses within short exposure periods, whereas reported or predicted concentrations of engineered nanomaterials in natural waters are generally much lower and vary with material type, source, aggregation, sedimentation, and transformation. For example, measured nanoparticle concentrations in surface waters can range from ng/L levels for Ag and μg/L levels for TiO_2_ [121,122]. Therefore, high-dose studies are useful for identifying toxic mechanisms, but they should not be directly used to define ecological risk thresholds. Future studies should include measured exposure concentrations, low-dose chronic exposure, and realistic water matrices containing natural organic matter, ions, and coexisting pollutants.

## 5. Methodological Advances for Cross-Scale Characterization of Nanomaterial-Microcystis Interactions

An important future direction in nanomaterial-Microcystis research is the transition from conventional bulk endpoints to a cross-scale characterization framework integrating near-native nanoparticle imaging, bio-interface-resolved spectroscopy, single-cell Raman phenotyping, particle/ion discrimination by Inductively Coupled Plasma Mass Spectrometry (ICP-MS), and multi-omics-based pathway mapping. To better connect methodology with toxicological mechanisms, these techniques should be selected according to the specific biological process being examined. Microscopy and elemental mapping are useful for distinguishing surface attachment, aggregation, and true internalization. Raman, Fourier Transform Infrared Spectroscopy (FTIR), and X-ray Photoelectron Spectroscopy (XPS) can reveal changes in lipids, proteins, polysaccharides, and surface functional groups related to membrane damage, EPS responses, and photosynthetic inhibition. ICP-MS-based methods help distinguish dissolved ion toxicity from particle-associated accumulation. Multi-omics approaches further link phenotypic toxicity with oxidative stress, photosynthesis, nutrient metabolism, and stress-response pathways.

### 5.1. Experimental Artifacts in Nanomaterial–Microalgae Toxicity Assays

Experimental artifacts are a key source of uncertainty in nanomaterial–microalgae toxicity assays. Aggregation and sedimentation can make the actual suspended dose much lower than the nominal concentration, especially for unstable or high-density particles. Particle shading may reduce light availability and photosynthetic activity, which should not be simply interpreted as direct cellular toxicity. In ROS and fluorescence-based assays, nanomaterials may adsorb probes, scatter light, quench fluorescence, or catalyze probe oxidation, leading to biased signals. In addition, particles attached to cell walls or extracellular polymeric substances can be mistaken for internalized particles. Therefore, exposure-medium characterization, particle stability analysis, proper blank and washing controls, and complementary imaging or elemental mapping are necessary to distinguish physical artifacts from true biological responses.

### 5.2. Microscopy and Imaging of Nano-Bio Interactions

Microscopy-based approaches remain essential for visualizing the physical interactions between nanomaterials and algal cells. Conventional transmission electron microscopy (TEM) and scanning electron microscopy (SEM) have been widely used to observe nanoparticle attachment, cell wall deformation, membrane disruption, intracellular accumulation, and ultrastructural damage [123]. However, these methods usually require dehydration, fixation, embedding, or sectioning, which may introduce artifacts and limit the interpretation of nano-bio interactions under native aqueous conditions.

Cryo-soft X-ray tomography (cryo-SXT) is a representative method for near-native three-dimensional imaging [124]. Unlike conventional electron microscopy, cryo-SXT preserves cells in a hydrated state by rapid cryofixation [125]. Within the water-window energy range, cellular components can be visualized based on their intrinsic X-ray absorption contrast, allowing three-dimensional reconstruction of intact and unstained cells with nanometer-scale spatial resolution [126]. This technique is particularly useful for determining whether nanoparticles remain attached to extracellular polymeric substances (EPS), accumulate on the cell surface, or enter the cytoplasm. More importantly, it allows nanoparticle localization to be interpreted together with organelle remodeling and physiological responses within the same cellular context.

For example, Xu et al. used cryo-SXT to investigate the interaction between citrate-coated AgNPs and *Chlamydomonas* reinhardtii under near-native conditions. After exposure to 1 mg/L AgNPs for 48 h, AgNPs were mainly distributed within EPS, while only a small fraction entered the cytoplasm. This indicates that EPS acts as an important interfacial region for the initial interaction between nanoparticles and algal cells. Three-dimensional quantitative analysis further showed that AgNP exposure induced pronounced subcellular remodeling, including decreases in chlorophyll a and b, disruption of chloroplast membrane structures, reduction in starch sheath thickness and pyrenoid volume fraction, and enlargement and redistribution of starch granules. These findings suggest that near-native imaging can provide evidence not only for “where nanoparticles are located”, but also for how nanoparticle exposure is associated with organelle damage and metabolic redistribution. This methodological framework is highly relevant for future studies on Microcystis, especially because EPS, mucilage layers, and colony structure may strongly regulate nanoparticle attachment, aggregation, and bioavailability [127].

In addition to cryo-SXT, hyperspectral imaging and enhanced dark-field microscopy can provide complementary information on nanoparticle detection and semi-quantification. Hyperspectral imaging combines spatial and spectral information, allowing researchers to correlate cellular morphology with material-specific optical signatures. It has been applied to detect and monitor nanoparticles in biological matrices using spectral libraries, which may be useful for distinguishing particle types and tracking their association with extracellular secretions or cell surfaces.

### 5.3. Raman-Based and Other Spectroscopic Tools for Single-Cell Biochemical Profiling

Spectroscopic techniques provide a non-destructive route to connect nanomaterial exposure with biochemical changes in algal cells. Compared with conventional endpoints, spectroscopic methods can detect changes in proteins, lipids, carbohydrates, pigments, nucleic acids, and surface functional groups. This is particularly important for *Microcystis*, because nanomaterials may affect not only cell growth and photosynthesis, but also EPS composition, toxin-related metabolism, and stress adaptation.

Raman spectroscopy is especially valuable for single-cell biochemical phenotyping [128,129]. It can provide molecular fingerprints of individual cells and detect changes in pigments, lipids, proteins, and carbohydrates without extensive sample preparation [129]. When combined with fluorescence mapping or mass spectrometry, Raman-based analysis can further link cellular biochemical heterogeneity with spatially resolved molecular information. Such approaches have been reported for single algal cell analysis and are promising for distinguishing subpopulations of *Microcystis* cells with different stress states under nanomaterial exposure.

FTIR spectroscopy and XPS can further resolve biointerface-level chemical changes. FTIR detects molecular vibrations and can identify alterations in functional groups related to polysaccharides, proteins, lipids, and cell wall components [130]. In algal toxicity studies, FTIR has been used to detect membrane damage and functional group modifications caused by ZnO nanoparticles in *Chlorella vulgaris* [131]. When combined with XPS, it can provide information on elemental composition and chemical bonding states, thereby helping to identify whether hydroxyl, carboxyl, amide, phosphate, or other functional groups participate in nanoparticle binding.

This strategy is also useful for interpreting EPS-mediated interactions. In a fungi-microalgae co-culture system under Cd stress, FTIR and XPS analyses showed that functional groups such as hydroxyl, amide, carbonyl, and phosphorus-containing groups participated in metal binding and adsorption [132]. SEM-EDX and TEM-EDX further revealed that metal accumulation occurred mainly through extracellular adsorption, while EPS production increased as a protective and detoxification response. Although this study focused on Cd rather than engineered nanoparticles, the same analytical logic can be applied to *Microcystis* systems to determine how EPS, cell surface chemistry, and nanoparticle coatings jointly regulate bioavailability and toxicity.

### 5.4. Multi-Omics Approaches for Pathway-Level Mechanistic Interpretation

Multi-omics approaches are becoming increasingly important for moving beyond phenotype-level toxicity assessment. Transcriptomics, metabolomics, proteomics, and lipidomics can identify stress-responsive pathways that are not apparent from growth inhibition or chlorophyll measurements alone. For nanomaterial-exposed *Microcystis*, these approaches are particularly useful for linking oxidative stress, photosynthetic inhibition, nitrogen metabolism, carbon fixation, membrane remodeling, EPS production, and microcystin-related metabolism.

Metabolomics provides direct information on small-molecule changes in algal cells. Recent metabolomics-based studies have shown that heavy metal stress can alter amino acids, fatty acids, carbohydrates, and antioxidant-related metabolites in microalgae. Time-series metabolomics further allows researchers to distinguish early stress responses from later acclimation processes. For example, under Cd stress, microalgal cells showed changes in chlorophyll content, MDA, GSH, and SOD, accompanied by EPS overproduction and metabolic shifts toward storage lipids and polysaccharide biosynthesis. These results demonstrate that metabolomics can reveal both short-term damage responses and longer-term adaptive regulation.

For *Microcystis*, metabolomics is particularly suitable for evaluating combined stress scenarios. A recent study investigated the responses of *Microcystis aeruginosa* to UV-B radiation and TiO_2_ nanoparticles in monoculture and in co-culture with *Lemna minor*. The results showed that growth was reduced under stress treatments, while co-culture conditions modified the overall response. Metabolomic profiling revealed treatment-specific changes in amino acid, lipid, organic acid, carbohydrate, and secondary metabolic pathways, suggesting that abiotic stressors and biological interactions can jointly reshape the metabolic state and oxidative stress response of *Microcystis*. Because this work is currently available as a preprint, its conclusions should be interpreted cautiously, but it still highlights the value of metabolomics for studying environmentally realistic nanomaterial exposure scenarios.

Metabolomics can also reveal species-specific mechanisms of nanoparticle toxicity. For instance, a recent AgNP study comparing *Euglena* sp. and *Navicula* sp. showed that AgNP toxicity in *Navicula* sp. was mainly associated with released Ag^+^ and oxidative stress-related metabolic changes, whereas energy metabolism disruption appeared to be more important in *Euglena* sp. The study also suggested that AgNP exposure may affect RNA biosynthesis pathways. These findings indicate that omics approaches can detect hidden molecular perturbations even when conventional oxidative damage endpoints are not obvious.

### 5.5. Particle-Specific Quantification and Speciation Analysis

A major challenge in nanomaterial ecotoxicology is distinguishing particulate toxicity from ion-mediated toxicity. Many metal and metal oxide nanoparticles undergo dissolution, aggregation, transformation, or surface complexation in aquatic media. Therefore, total metal concentration alone is insufficient for identifying the actual toxic species that interact with *Microcystis* cells. Particle-specific quantification and speciation analysis are needed to determine whether toxicity is driven by intact particles, released ions, transformed species, or particle-cell aggregates.

ICP-MS-based techniques are particularly useful in this context. HPLC-ICP-MS can separate and quantify different elemental species, helping to distinguish nanoparticles from low-molecular-weight metal species. Single-cell ICP-MS further enables quantification of metal uptake at the individual-cell level rather than relying only on average bulk concentrations. These methods are valuable for evaluating heterogeneous exposure among algal cells, especially in colonial or aggregated *Microcystis* populations where nanoparticle distribution may be uneven.

Recent AgNP studies illustrate why this distinction is important. In AgNP exposure experiments, ICP-MS has been used to determine intracellular Ag accumulation and Ag^+^ release, allowing researchers to compare particle-associated toxicity with dissolved ion toxicity. Evidence from different algal species indicates that the contribution of Ag^+^ release, cell-associated Ag accumulation, and nanoparticle morphology can vary substantially with algal cell structure and exposure conditions. For example, morphology-dependent studies showed that silver nanoplates, nanocubes, and nanospheres differed in colloidal stability, cellular damage patterns, and species-specific toxicity toward *Chlorella vulgaris* and *Scenedesmus obliquus*. These findings support the need to integrate particle characterization, cellular accumulation analysis, and biological endpoints when assessing nanoparticle risks.

## 6. Conclusions and Prospects

Engineered nanomaterials can exert diverse toxic effects on microalgae through both particle-specific and material-dependent mechanisms. Metal and metal oxide nanoparticles mainly affect algal cells through surface attachment, toxic ion release, membrane damage, oxidative stress, photosynthetic inhibition, and intracellular disturbance. Nanoplastics can interact with algal cell surfaces, promote heteroaggregation and internalization, and induce ROS overproduction, membrane permeability changes, and growth inhibition. Carbon-based nanomaterials, including graphene-family materials, carbon nanotubes, and carbon nanofibers, may impair microalgae through shading effects, nutrient adsorption, mechanical damage, oxidative stress, and disruption of cellular metabolism. Although different nanomaterials show distinct toxicity patterns, growth inhibition, chlorophyll reduction, photosynthetic impairment, oxidative stress, membrane injury, and metabolic disturbance are common toxic responses.

The toxicity of nanomaterials to microalgae is strongly regulated by both intrinsic physicochemical properties and environmental conditions. Particle size, morphology, surface coating, dissolution, ion release, aggregation, and aging determine nanomaterial stability, bioavailability, and interaction with algal cells. Environmental factors such as light, pH, and natural organic matter further modify nanoparticle transformation, surface charge, ROS generation, cellular uptake, and toxicity. These factors may either enhance or reduce toxicity depending on the material type, exposure concentration, algal species, and surrounding water chemistry. Therefore, toxicity should be understood as a dynamic process rather than a fixed material property. A universal safe concentration for engineered nanomaterials in microalgae cannot be defined at this stage. Toxicity thresholds are material- and scenario-dependent and should be established using measured exposure concentrations, long-term low-dose tests, and environmentally realistic co-exposure systems.

Under environmentally realistic conditions, engineered nanomaterials rarely occur as single stressors. Their coexistence with other nanoparticles, natural colloids, pharmaceuticals, antibiotics, pesticides, disinfectants, and organic pollutants can lead to synergistic, antagonistic, additive, or concentration-dependent effects. These combined effects are largely controlled by changes in particle aggregation, dissolution, pollutant adsorption, cellular uptake, EPS secretion, and oxidative stress responses. Thus, future risk assessment should move beyond single-material exposure systems and pay more attention to multi-stressor scenarios that better represent natural aquatic environments.

Future studies should further integrate advanced characterization methods with biological mechanism analysis. Near-native imaging, hyperspectral imaging, Raman spectroscopy, FTIR, XPS, ICP-MS-based particle/ion discrimination, single-cell analysis, and multi-omics approaches can provide cross-scale information from nanoparticle behavior and nano-bio interface interactions to cellular damage and pathway-level responses. Such integrated methods are particularly important for clarifying how nanoparticles interact with Microcystis and other bloom-forming algae, how EPS and colony structures regulate bioavailability, and how nanomaterials influence photosynthesis, metabolism, oxidative stress, and toxin-related processes. Overall, developing more realistic exposure systems, improving particle-specific characterization, and linking material transformation with algal physiological and molecular responses will be essential for understanding the ecological risks of engineered nanomaterials in aquatic environments.

## Data Availability

No new data were created or analyzed in this study. Data sharing is not applicable to this article.
